# Association of Cardiovascular Risk Factors with Carotid Intima Media Thickness in Patients with Rheumatoid Arthritis with Low Disease Activity Compared to Controls: A Cross-Sectional Study

**DOI:** 10.1371/journal.pone.0140844

**Published:** 2015-10-20

**Authors:** Deborah F. van Breukelen—van der Stoep, Derkjen van Zeben, Boudewijn Klop, Gert-Jan M. van de Geijn, Hans J. W. Janssen, Mieke J. M. W. Hazes, Erwin Birnie, Noelle van der Meulen, Marijke A. De Vries, Manuel Castro Cabezas

**Affiliations:** 1 Department of Rheumatology, Sint Franciscus Gasthuis, Rotterdam, The Netherlands; 2 Department of Internal Medicine, Sint Franciscus Gasthuis, Rotterdam, The Netherlands; 3 Department of Clinical Chemistry, Sint Franciscus Gasthuis, Rotterdam, The Netherlands; 4 Department of Rheumatology, Erasmus Medical Centre, Rotterdam, The Netherlands; 5 Institute of Health Policy and Management, Erasmus University, Rotterdam, The Netherlands; 6 Statistics and Education, St. Franciscus Gasthuis, Rotterdam, The Netherlands; Rutgers University, UNITED STATES

## Abstract

**Objectives:**

Rheumatoid arthritis (RA) has been identified as an independent cardiovascular risk factor. The importance of risk factors such as hypertension and hyperlipidemia in the generation of atherosclerosis in RA patients is unclear. This study analyzed clinical parameters associated with carotid intima media thickness (cIMT) in patients with RA.

**Methods:**

Subjects with RA and healthy controls without RA, both without known cardiovascular disease, were included. Participants underwent a standard physical examination and laboratory measurements including a lipid profile. cIMT was measured semi-automatically by ultrasound.

**Results:**

In total 243 RA patients and 117 controls were included. The median RA disease duration was 7 years (IQR 2–14 years). The median DAS28 was 2.4 (IQR 1.6–3.2) and 114 (50.4%) of the RA patients were in remission. The presence of RA and cIMT were not associated (univariate analysis). Multivariable regression analysis showed that cIMT in RA patients was associated with age (B = 0.006, P<0.001) and systolic blood pressure (B = 0.003, P = 0.003). In controls, cIMT was associated with age (B = 0.006, P<0.001) and smoking (B = 0.097, P = 0.001).

**Conclusion:**

cIMT values were similar between RA patients and controls. Hypertension was strongly associated with cIMT in RA patients. After adjustment, no association between cIMT and specific RA disease characteristics was found in this well treated RA cohort.

## Introduction

Rheumatoid Arthritis (RA) is a chronic systemic inflammatory disease of unknown etiology, affecting ~1% of the adult general population [1, 2]. Patients with RA have a higher all cause mortality risk than the general population and the leading cause of death is cardiovascular disease (CVD) [3–5]. The evidence of the excess cardiovascular risk in rheumatoid arthritis (RA) has been well described [3, 5–9] and the presence of RA can be considered an independent risk factor for CVD [7, 10]. The prevalence of CVD in patients with RA is as high as in patients with type 2 diabetes mellitus [7, 11]. Since inflammation is a key event in the development of atherosclerosis [12–14] it has been proposed that the increased inflammatory state of patients with RA explains, at least in part, the increased cardiovascular risk [8, 10, 15–17]. Several RA specific risk factors such as disease activity, inflammatory markers and anti-CCP have been associated with an increased carotid intima media thickness (cIMT) and CVD risk. A recent study by Barbarroja et al. showed that anti-CCP antibodies act as direct inductors of the pro-oxidative status and the inflammatory and atherogenic profile of lymphocytes, monocytes and neutrophils in RA [18]. In line with this report, Vázquez et al. showed an association between anti-CCP levels and CRP levels with an increased cIMT and CVD risk [19]. Moreover, traditional risk factors such as hypertension, hyperlipidemia, smoking and overweight are also highly prevalent among RA patients [20, 21], and several studies have shown a significant underdiagnosis and undertreatment of these traditional risk factors in RA [22, 23].

The cIMT measured by ultrasound is a surrogate marker of atherosclerosis and the most widely used non invasive imaging method to assess atherosclerosis and CVD risk. A higher cIMT reflects a (pre-) atherogenic condition and is predictive for future cardiovascular events [24, 25]. Several studies have shown an increased cIMT in RA patients, even early in the course of the disease [26–30]. Traditional risk factors may also play a role besides the inflammatory state in RA. Unfortunately, the association between traditional risk factors and cIMT in RA patients is still unclear [31]. Increasing evidence suggests that a cumulative number of traditional CVD risk factors contributes to the higher CVD risk in RA [32]. The aim of this study was to investigate which factors are associated to cIMT in RA patients in comparison to controls.

## Materials and Methods

### Study design and subjects

A cross-sectional study was carried out in patients with RA and controls to determine the differences in the relationship between cIMT and clinical factors. The study was carried out between July 2009 and February 2013 at the Diabetes and Vascular Centre and the outpatient clinic of Rheumatology of the Sint Franciscus Gasthuis, Rotterdam, the Netherlands. All patients with RA in this report were participants in the FRANCIS study, an open label randomized clinical trial to investigate the effectiveness of strict treatment of cardiovascular risk factors in RA (The Dutch Trialregister, NTR3873; ABR no. NL32669.101.10). RA patients attending the outpatient clinic from the Department of Rheumatology were asked to participate in the FRANCIS study. Inclusion criteria were the presence of RA and an age ≤70 years. Exclusion criteria were the presence of diabetes mellitus (DM) or CVD. CVD was defined as a documented history of myocardial infarction, cerebrovascular event, amputation due to peripheral artery disease, intermittent claudication, or a prior percutaneus transluminal coronary angioplasty (TCA) or coronary artery bypass graft (CABG). In addition, kidney disease defined as an MDRD< 40 was an exclusion criterium. Only patients with a CVD risk <10% according to the 2009 version of the SCORE model were randomized. Patients aged over 65 years were classified as 65 years old in order to be able to use the SCORE model. Patients with a CVD risk ≥10% were followed in a separate cohort (“high risk cohort”). For the current analysis, baseline data from both randomized patients and patients in the high risk cohort were used. The RA patients were treated by their own rheumatologists according to a treat-to-target principle, aiming for disease remission (DAS28<2.6). RA was defined by the ACR ‘87 criteria [33]. Unmatched control subjects were non-RA patients followed in a separate observational study (ABR no. NL29910.101.09) from our department. They were recruited from the outpatient clinic of the Diabetes and Vascular Center of the Sint Franciscus Gasthuis in Rotterdam and underwent measurements identical to the RA patients of the FRANCIS study [34]. Exclusion criteria for the control group were also the presence of DM, CVD and/or kidney disease. All controls fulfilling the age limit of ≤70 years were included in this analysis. In addition, RA patients and controls who used statins and/or anti-hypertensives were excluded. Controls with a CRP >10 mg/L were excluded from the analysis. Anthropometric characteristics i.e. height, weight, waist circumference and blood pressure were obtained as well as a detailed medical history and the use of medication. All subjects provided written informed consent. The studies were approved by the independent Regional Medical Ethical Committee Rotterdam of the Maasstad Hospital, the Netherlands.

### Rheumatoid arthritis disease activity

Rheumatoid arthritis disease activity was assessed by using the Disease Activity Score with 28 joints counted (DAS28). This score included swollen joint count (28), tender joint count (28), VAS (0–100 scale) indicating pain/discomfort due to RA, and the erythrocyte sedimentation rate (ESR). We also calculated disease activity with C-reactive protein (CRP) instead of ESR (DAS28CRP),

### Laboratory measurements

A standardized set of measurements was performed in each subject. Laboratory parameters were determined at the Department of Clinical Chemistry, Sint Franciscus Gasthuis, according to standard procedures. Renal and liver function tests as well as glucose, CRP, total cholesterol, HDL-C and triglycerides (TG) were measured using Synchron LX-20 and DxC analyzers (Beckman Coulter, Anaheim CA, USA) (34, 35). LDL-C was calculated using the Friedewald formula if TG were below 4.60 mmol/l. Apolipoprotein (apo) AI and apo B were determined in serum by rate nephelometry using IMMAGE with commercially available kits (Beckman Coulter, Anaheim CA, USA) [31,32]. In the case of RA patients, blood samples were obtained following the strict protocol of the FRANCIS study. Samples from control subjects were drawn with less strict criteria.

### Intima media thickness of the carotid arteries

Carotid ultrasound scans were carried out using the ART-LAB (Esaote, Italy) by trained and experienced sonographers as described earlier [34, 35]. Ultrasound scans were performed with the patients lying in a supine position with the head resting comfortably and the neck slightly hyperextended and rotated in the opposite direction of the probe. The ultrasound images were obtained of the distal 1 cm of the far wall of each common carotid artery (CCA) using B-mode ultrasound producing two echogenic lines. These lines represent the combined thickness of the intima and media layers of the arterial wall. Each CCA was imaged in three different projections: CCA right side 90-120-150 and CCA left side 210-240-270 degrees. The segments were measured semi-automated in triplicate. In some patients cIMT measurements were only partly available due to technical difficulties. In these cases, the mean cIMT was only calculated if at least measurements at two different angles per side were available.

### Statistics

Data are given as mean ± standard deviation (SD) unless stated otherwise. Differences between groups were determined using the unpaired Student’s t-test or Chi-square test, where appropriate. In case of skewed variables (DAS28(-CRP) and TG), the Mann-Whitney U test was used for comparisons between groups. For statistical analysis, cIMT was defined as the mean of the six individual measurements. Multivariable linear regression analysis was used to identify factors associated with cIMT in RA patients and controls. Covariables entered into the regression analysis were: age, sex, systolic blood pressure, waist circumference, smoking habit, glucose, apo AI, apo B, total cholesterol, HDL-C, LDL-C, TG, glucose, creatinin and CRP. For RA patients, the presence of rheumatoid factor and anti-CCP, erosive disease, disease duration, DAS28CRP, DAS28 and the use of methotrexate and biologicals were added to the model. Covariables showing a P<0.10 in the univariate analysis were entered in the multivariable analysis. In order to create comparable models for RA patients and controls, a separate analysis with and without RA disease duration was performed for RA patients. In the case that two or more related parameters were highly correlated (multicollinearity), only one was selected for the multivariable analysis. As a result, waist circumference was selected instead of BMI; systolic blood pressure was selected instead of diastolic blood pressure; and LDL-C was chosen above apo B or TG. Significant co-variables were investigated for interaction. The multivariable analysis included age, sex, smoking, waist circumference, systolic blood pressure and LDL-C. The association between glucose and cIMT was analyzed separately because it was unknown which of the control subjects had their blood samples taken after an overnight fast. All statistical analyses were performed using PASW statistics version 18.0 (IBM SPSS Statistics, New York, United States). P-values below 0.05 (two sided) were considered statistically significant.

## Results

### General Characteristics

243 RA patients and 117 controls were included. General characteristics of RA patients and controls are shown in Table 1. Both groups were comparable regarding age, gender, lipid profile, waist circumference, and cIMT. There were more current smokers among RA patients compared to controls. RA patients had a significantly higher body mass index (BMI) and systolic blood pressure (Table 1).

**Table 1:** General Characteristics.

	RA-patients	Controls	P-value
	n = 243	n = 117	
Male	79 (32.5%)	46 (39.3%)	0.204
Age (yrs)	51.8±11.6	50.0±12.1	0.186
Current smoker (n,%)	54 (22.2%)	16 (13.7%)	0.051
Weight (kg)	76.3±14.4	75.3±14.5	0.520
BMI (kg/m^2^)	26.1±4.3	24.9±3.7	0.006
Systolic blood pressure (mmHg)	129±18	124±13	0.004
Waist circumference (cm)	93±14	90±12	0.054
Total cholesterol (mmol/l)	5.42±1.08	5.60±1.17	0.147
LDL-C (mmol/l)	3.36±1.02	3.46±1.00	0.372
HDL-C (mmol/l)	1.54±0.51	1.55±0.43	0.894
TG (mmol/l)*	1.03 (0.72–1.42)	1.12 (0.74–1.61)	0.182
Apo AI (g/l)	1.71±0.36	1.65±0.32	0.099
Apo B (g/l)	1.00±0.26	1.04±0.30	0.192
Glucose (mmol/l)	5.40±0.53	5.16±0.89	0.002
C-reactive protein (mg/l)*	2.00 (1.00–5.00)	1.00 (1.00–2.75)	<0.001
cIMT (mm)	0.556±0.120	0.573±0.134	0.220
Rheumatoid Factor Positive (n,%)	150 (61.7%)**	NA	
Anti-CCP positivity (n,%)	142 (58.4%)***	NA	

BMI = body mass index TG = triglycerides; Apo = apolipoprotein; cIMT = carotid intima media thickness

Data presented as mean ± standard deviation or n (%) unless stated otherwise.

* Data presented as median (interquartile range). Difference between groups tested with the Mann-Whitney U test.

** data available in 235 patients.

*** data available in 204 patients

NA, not available

The median RA disease duration for RA patients was 7 years (IQR 2–14 years). In total, 107 patients (44.0%) used NSAIDS, 186 (76.5%) RA patients used methotrexate and 96 (39.5%) used an anti-TNFα or other biological agent. Of the 96 patients with a biological agent, 25 (26%) used no methotrexate and 71 (74%) used a combination of methotrexate and a biological.

The median DAS28 was 2.4 (IQR 1.6–3.2). In total, 114 (50.4%) of the RA patients had a DAS28 <2.6 implying disease remission, 48 (21.2%) had low disease activity (DAS28 2.6–3.1), 47 (20.7%) had moderate disease activity (DAS28 3.1–5.1) and 17 (7.5%) had high disease activity (DAS28 ≥ 5.1). When applying DAS28CRP, the results were similar: the median DAS28CRP was 2.2 (IQR 1.6–2.9), 113 (58.8%) had disease remission, 39 (17.3%) had low disease activity, 40 (17.7%) had moderate disease activity and 14 (6.2%) had high disease activity. cIMT did not differ between RA groups based on their disease activity (data not shown).

### Univariate analysis

To identify the covariables associated with cIMT, univariate regression analysis was carried out in RA patients, controls and the combined patient and control group (Table 2). In all groups age, systolic blood pressure, LDL-C and apo B showed a significant association with cIMT. Furthermore, in controls, current smoking was significantly associated with cIMT. In RA patients male gender, BMI, waist circumference, TG, glucose and disease duration were significantly associated with cIMT. The correlation coefficient between RA disease duration and age was 0.262. A trend towards a positive association between anti-CCP positivity and cIMT was observed (B = 0.033(-0.044–0.070); P = 0.078) (Table 2). When these univariate analyses were performed only in RA patients not using biologicals, the associations between cIMT and anti-CCP positivity, disease duration, glucose and TG were no longer statistically significant (data not shown). Other parameters showed similar associations as in the total RA group.

**Table 2:** Univariate regression analysis identifying covariables associated with cIMT.

Variable	Controls n = 117		RA patients n = 243		Total population n = 360	
	B (95% CI)	P-value	B (95% CI)	P-value	B (95% CI)	P-value
Age (yr)	0.006 (0.004–0.007)	<0.001	0.006 (0.005–0.007)	<0.001	0.006 (0.005–0.007)	<0.001
Male sex	0.021 (-0.029–0.071)	0,243	0.050 (0.018–0.082)	0.002	0.041 (0.014–0.068)	0.003
Smoking	0.097 (0.028–0.166)	0.006	0.016 (-0.021–0.053)	0.403	0.034 (0.001–0.067)	0.044
BMI (kg/m^2^)	0.005 (-0.002–0.012)	0.139	0.005 (0.001–0.008)	0.010	0.004 (0.001–0.007)	0.006
Waist (cm)	0.002 (0.000–0.004)	0.101	0.002 (0.001–0.003)	<0.001	0.002 (0.001–0.003)	<0.001
BPs (mmHg)	0.002 (0.001–0.004)	0.009	0.003 (0.002–0.004)	<0.001	0.003 (0.002–0.003)	<0.001
BPd (mmHg)	0.000 (-0.003–0.003)	0.963	0.004 (0.002–0.005)	<0.001	0.003 (0.001–0.004)	<0.001
LDL-C (mmol/l)	0.043 (0.019–0.066)	<0.001	0.034 (0.020–0.049)	<0.001	0.037 (0.025–0.050)	<0.001
HDL-C (mmol/L)	-0.010 (-0.066–0.047)	0.740	-0.010 (-0.040–0.020)	0.511	-0.010 (-0.037–0.017)	0.476
Apo AI (g/l)	0.013 (-0.065–0.091)	0.750	-0.016 (-0.059–0.027)	0.473	-0.010 (-0.048–0.028)	0.610
Apo B (g/l)	0.114 (0.034–0.194)	0.005	0,131 (0.075–0.187)	<0.001	0.126 (0.081–0.172)	<0.001
TG (mmol/l)*	0.013 (-0.020–0.045)	0.441	0.026 (0.003–0.049)	0.026	0.022 (0.003–0.040)	0.022
Glucose (mmol/l)*	0.009 (-0.019–0.036)	0.536	0.044 (0.016–0.072)	0.002	0.021 (0.001–0.040)	0.029
CRP (mg/dl)	-0.000 (-0.014–0.014)	0.964	0.001 (-0.001–0.003)	0.504	0.000 (-0.002–0.002)	0.731
Creatinin (umol/l)	0.001 (-0.001–0.003)	0.549	0.002 (0.001–0.003)	0.002	0.001 (0.001–0.003)	0.002
Absence of RA	-	-	-	-	0.017 (-0.010–0.045)	0.220
Anti-CCP	-	-	0.033 (-0.004–0.070)	0.078		
Rheumatoid factor	-	-	0.020 (-0.013–0.053)	0.231		
Erosive Disease	-	-	0.020 (-0.011–0.051)	0.212		
DAS28	-	-	0.005 (-0.009–0.019)	0.494		
DAS28CRP	-	-	-0.001 (-0.017–0.015)	0.902		
Disease duration	-	-	0.002 (0.001–0.004)	0.012		
Methotrexate	-	-	0.001 (-0.036–0.037)	0.976		
Biological agents	-	-	-0.07 (-0.039–0.024)	0.651		

RA = Rheumatoid arthritis, BPs = systolic blood pressure, BPd = diastolic blood pressure, TG = triglycerides, DAS28 = RA disease activity score 28 joints

### Multivariable analysis

Multivariable regression analysis with identified covariables associated with cIMT, was performed in RA patients, controls and in both groups combined (Table 3). In controls cIMT was significantly associated with age and current smoking and in RA patients with age and systolic blood pressure. These results remained unchanged after including glucose in the analysis. In RA patients, additional multivariable analyses including RA disease duration alone and combined with anti-CCP were performed (Table 4). The addition of RA disease activity and anti-CCP positivity, alone or combined, did not improve the predictive power of the original multivariable model (Table 4). Multivariable analysis in only those RA patients not using biological therapy showed comparable results.

**Table 3:** Multivariable regression analysis with identified covariables associated with cIMT.

Variable	Controls n = 117		RA patients n = 243		Total population n = 360	
	R^2^ = 0.321		R^2^ = 0.391		R^2^ = 0.347	
	B (95% CI)	P-value	B (95% CI)	P-value	B (95% CI)	P-value
Age (yr)	0.005 (0.003–0.007)	<0.001	0.005 (0.004–0.006)	<0.001	0.005 (0.004–0.006)	<0.001
Male sex	0.010 (-0.037–0.056)	0.678	0.013 (-0.014–0.040)	0.336	0.015 (-0.008–0.038)	0.201
Smoking	0.101 (0.041–0.162)	0.001	0.019 (-0.011–0.049)	0.207	0.034 (0.007–0.061)	0.014
Waist (cm)	0.002 (0.000–0.004)	0.127	0.001 (0.000–0.002)	0.142	0.001 (0.000–0.002)	0.106
BPs (mmHg)	0.001 (-0.001–0.002)	0.488	0.001 (0.000–0.002)	0.003	0.001 (0.000–0.002)	0.012
LDL-C (mmol/l)	0.009 (-0.016–0.034)	0.467	0.007 (-0.006–0.020)	0.270	0.009 (-0.003–0.020)	0.143
Apo B (g/l)		**		**		**

RA = Rheumatoid arthritis, BPs = systolic blood pressure, Apo = apolipoprotein

** When using apoB instead of LDL similar results were found.

**Table 4:** Multivariable regression analysis including RA disease characteristics.

Variable	RA patients n = 243		RA patients n = 243	
	R^2^ = 0.39		R^2^ = 0.40	
	B (95% CI)	P-value	B (95% CI)	P-value
Age (yr)	0.005 (0.004–0.006)	<0.001	0.005 (0.004–0.007)	<0.001
Male sex	0.005 (-0.016–0.039)	0.409	0.005 (-0.026–0.035)	0.786
Smoking	0.019 (-0.011–0.049)	0.212	0.020 (-0.02–0.053)	0.220
Waist (cm)	0.001 (0.000–0.002)	0.156	0.001 (0.000–0.002)	0.216
BPs (mmHg)	0.001 (0.000–0.002)	0.002	0.001 (0.000–0.000)	0.005
LDL-C (mmol/l)	0.007 (0.006–0.020)	0.282	0.007 (-0.008–0.021)	0.365
RA disease duration (yrs)	0.000 (-0.002–0.001)	0.579	0.000 (-0.002–0.002)	0.792
Anti-CCP	-	-	0.007 (-0.024–0.037)	0.661

RA = Rheumatoid Arthritis, BPs = systolic blood pressure

## Discussion

Surprisingly, cIMT in RA patients was similar to the cIMT of control subjects without RA, which is in contrast to other studies, showing increased cIMT in RA patients even early in the course of the disease [29, 30, 32, 36–38]. It is even more surprising since in our study RA patients had a higher BMI, systolic blood pressure, glucose and CRP and, a larger percentage of RA patients smoked compared to controls.

A post hoc sample size calculation showed that our sample sizes of 243 RA-patients and 117 control patients are not sufficiently large to support the presence of a significant difference in mean cIMT between the groups with 80% power. However, considering that the difference in mean cIMT we found (0.017) cannot be considered clinically meaningful, one would need to include (at least) 270 RA and control patients (at an equivalence margin of 0.05; or 68 patients at an equivalence margin of 0.10) to support that a difference in cIMT is absent. In summary, our sample sizes are sufficiently large to support that a clinically meaningful difference in cIMT in our study is absent, with 90% power.

One of the reasons for this difference with other studies may be the relatively low disease activity in our RA patients. A higher RA disease activity results in an increased cardiovascular risk. The RA disease activity in our study was low with a total of 70% of RA patients in clinical remission or with low disease activity, which may have had a beneficial effect on cIMT. When we compared cIMT in RA patients with disease remission or low disease activity with cIMT in RA patient with moderate or high disease activity, no differences in cIMT were found. This may be due in part to the small proportion of patients with moderate to high disease activity. Our outpatient clinic of Rheumatology is a large outpatient clinic that consists of a large and representative sample of RA patients in the Netherlands. At the outpatient clinic, a structured tight control regimen of RA disease activity is carried out reflecting the present situation in the Netherlands. Therefore, we believe that the sample of RA patients in this study is representative for the general Dutch RA population, although the results may not apply to RA patients with higher average disease activity. A meta analysis by Ambrosino et al. reported outcomes of 59 studies on cIMT in RA compared to controls [30]. Not all studies included information on disease activity, but the mean DAS28 of those studies reporting disease activity ranged from 2.6 to 6.2, which is higher than our reported median DAS28 of 2.4. Of those 59 studies, 51 reported an increased cIMT in RA patients compared to controls [30]. Only 2 studies reported DAS28 levels <3.0 showing conflicting results concerning differences in cIMT in RA compared to controls [39, 40]. The same meta-analysis by Ambrosino showed that a higher inflammatory status (DAS28, CRP, ESR) was associated with an increased cIMT [30].

Besides disease activity, several RA specific risk factors, such as the presence of rheumatoid factor, anti-CCP and erosive disease, are considered risk factors for atherosclerosis. They are all surrogate markers for the inflammatory burden since the presence of reumatoid factor and/or anti-CCP often results in a higher disease activity. Also erosive disease occurs mostly in patients with long periods of uncontrolled disease activity. In our cohort with generally a low disease activity, rheumatoid factor and anti-CCP positivity were not associated with cIMT in the multivariable model. These results are in line with several other studies that could not demonstrate an association between these parameters and cIMT [37, 39]. A limitation of our study was that only qualitative data on rheumatoid factor and anti-CCP were available, since these characteristics were not re-tested at the time of inclusion. Recently, an *in vitro* study showed a positive correlation between the level of anti-CCPs and overexpression of thrombotic, inflammatory and pro-oxidative markers in leukocytes [18]. We did find a crude (univariate) association between disease duration and cIMT, but this association was no longer significant in the multivariable model. This is in line with a recent publication by Arts et al who found that the risk of CVD in RA patients was not increased after 10 years of disease duration when compared to the first 10 years [40]. Data on the association between cIMT and RA disease duration are conflicting since several other studies reported a positive association between cIMT and RA disease duration [37, 41, 42].

Traditional cardiovascular risk factors such as smoking, overweight, hyperlipidemia and hypertension are highly prevalent in RA patients and can be easily treated by lifestyle and pharmacological interventions. Nevertheless, underdiagnosis and undertreatment of these risk factors in RA patients have been described [22, 23, 43]. An association between cIMT and blood pressure both in the general population and in RA patients has been described previously [17, 27, 44–46]. In the present paper, regression analyses underline the association of systolic blood pressure on cIMT in RA patients. These data underscore the importance of adequate blood pressure regulation in RA.

No association between cIMT and lipid levels was found. Hyperlipidemia was strongly associated to cIMT in the univariate analysis, but it was no longer significant in the multivariable analysis after adjustment for other covariables. In contrast to the association found between smoking and cIMT in controls, no association between smoking and cIMT in RA patients was found. This is in line with earlier results reported by Gonzalez et al., who found a weaker association between smoking and CVD risk in RA patients than in non-RA patients [47].

In conclusion, in this RA cohort with relatively low disease activity, cIMT was not increased compared to controls and cIMT was not associated with RA disease characteristics. Hypertension was strongly associated with cIMT in RA patients. In light of these results and based on findings from other studies, treatment of hypertension and other traditional CVD risk factors in RA seems warranted to reduce cardiovascular risk in RA.
